# The Relationship Between Female Labor Supply and Caregiving Over the Life Cycle

**DOI:** 10.1093/geroni/igaa057.1956

**Published:** 2020-12-16

**Authors:** Alison Aughinbaugh

**Affiliations:** BLS, Washington, District of Columbia, United States

## Abstract

I examine the effects of caring for others on female labor supply over the life-cycle using a fixed effect model. The data come from the National Longitudinal Survey of Youth 1979 (NLSY79), which collects information about the care of each child during his first three years and the care provided to household members during a woman’s 50s. The NLSY79 data show that women’s labor supply drops around the time a child is born and then rises, with over 50 percent working by time their children reach age 2. In addition, these data show that during their 50s, about 9 percent of women provide care to someone living in their household and that these female caregivers spend about 40 hours per week providing care. Time spent in caregiving may affect time in the labor force, and hence the ability to invest in a career and accumulate work experience and wage growth.

